# Pradofloxacin for Treatment of *Bartonella henselae* in Experimentally Inoculated Cats

**DOI:** 10.3390/pathogens13040336

**Published:** 2024-04-18

**Authors:** Michael R. Lappin, Ronan Fitzgerald

**Affiliations:** 1 Center for Companion Animal Studies, Colorado State University, Fort Collins, CO 80523, USA; 2 Elanco Animal Health, Innovation Way, Greenfield, IN 46140, USA; rafitzgerald@btinternet.com

**Keywords:** pradofloxacin, *Bartonella*, neutropenia, lymphopenia

## Abstract

*Bartonella henselae* is associated with numerous clinical syndromes in people. Cats are the definitive hosts for *B. henselae*, develop high levels of bacteremia, and are associated with human infections, particularly in the presence of *Ctenocephalides felis*. Several antibiotic protocols used for the treatment of *B. henselae* infection in cats have failed to clear bacteremia. The purpose of this study was to assess the safety and efficacy of a high-dose pradofloxacin protocol to eliminate *B. henselae* bacteremia. *Bartonella henselae* infection was initiated in 8 cats by intravenous inoculation of infected feline blood and then pradofloxacin was administered at 7.5 mg/kg, PO, twice daily for 28 days, starting 12 weeks after inoculation. Complete blood cell counts were performed prior to pradofloxacin administration and then every 2 weeks for 10 weeks. *Bartonella* PCR assay was performed prior to pradofloxacin administration and approximately every 2 weeks for 10 weeks and then weekly for 4 weeks. Methylprednisolone acetate (5 mg/kg) was administered by intramuscular injection to all cats on week 10. The cats remained normal and none developed a hematocrit, platelet count, lymphocyte count, or neutrophil count outside of the normal reference ranges. In the one month prior to pradofloxacin administration, all cats were PCR-positive for *Bartonella* DNA on at least two of four sample dates; after pradofloxacin administration, all cats were negative for *B. henselae* DNA in blood on all nine sample dates. The protocol appears to be safe and failure to amplify *B. henselae* DNA from the blood after the administration of pradofloxacin and one dose of methylprednisolone acetate suggests either an antibiotic effect or the organism was cleared spontaneously.

## 1. Introduction

*Bartonella henselae* is the most common cause of cat scratch disease and other clinical syndromes including endocarditis and peliosis hepatis in people [1]. Cats develop high levels of *B. henselae* bacteremia which are associated with human infections, particularly in the presence of *Ctenocephalides felis*, likely as a result of contact with infected flea frass [1,2,3]. Multiple clinical and laboratory abnormalities have been associated with *Bartonella* spp. in some cats. Fever, cardiac abnormalities, and hyperglobulinemia are some of the most common [1,3,4,5,6,7,8,9]. Several antibiotic protocols used for the treatment of *B. henselae* infection in cats, including doxycycline, azithromycin, or enrofloxacin administered alone, have failed to clear bacteremia in treated cats [3,4,10,11]. This may relate to the intracellular phase of the organism and the spectrum, dose, or duration of the antibiotics that were prescribed. 

Pradofloxacin is a third-generation fluoroquinolone, approved for use in cats (and also for dogs in some countries) [12,13]. This fluoroquinolone displays dual targeting of two bacterial enzymes in contrast to the preferred target (DNA gyrase) of the other fluoroquinolones approved for cats in some countries (enrofloxacin, marbofloxacin, orbifloxicin) and so has increased activity against some bacteria. When grown in the presence of three antibiotics, *B. henselae* isolates from a human and a cat were less likely to develop resistance to pradofloxacin, when compared to azithromycin or enrofloxacin [14]. In addition, the administration of pradofloxacin has not been associated with the retinal degeneration in cats that has resulted from administration of some of the other quinolones [15]. However, in the United States, the pradofloxacin drug label (7.5 mg/kg, PO, once daily) states that some cats treated at 3X and 5X the label dose for 21 days showed evidence of reversible bone marrow abnormalities (https://animaldrugsatfda.fda.gov/adafda/app/search/public/document/downloadFoi/901) (accessed on 14 April 2023). 

Since *B. henselae* is a human pathogen, in some situations (such as in the homes of immunosuppressed people) it might be desirable to eliminate bacteremia in cats. While pradofloxacin has been used in combination with doxycycline in several case reports [6,7,8], no study has assessed the safety and efficacy of a high-dose pradofloxacin protocol to eliminate *B. henselae* from the blood of experimentally infected cats.

## 2. Materials and Methods

The study was approved by the Institutional Animal Care and Use Committee of a contract research facility (protocol # 170.029) and was monitored by the United States Department of Agriculture. In a separate study, infection with the CSU Bh1 strain of *B. henselae* was initiated in 8 young adult (12–16 months of age), mixed sex (4 male; 4 female), specific pathogen-free cats by intravenous inoculation of 1 mL of infected feline blood over 1 min; the number of organisms administered was unknown. A total of 12 weeks after inoculation, the cats were transferred to the study described here. Before *B. henselae* inoculation, these healthy cats had been housed in a vector-free research facility without flea control since birth and were negative for feline leukemia virus antigen, feline immunodeficiency virus antibodies, and *Bartonella* spp. antibodies, as well as *Bartonella* spp. DNA and hemoplasma DNA by PCR assay. In both studies, *Bartonella* spp. antibodies were detected by a previously reported ELISA using *B. henselae* as the antigen source and *Bartonella* spp. DNA was amplified as previously described [3,4].

This strain of *B. henselae* had previously been tested for pradofloxacin susceptibility, using the E-test in the laboratory of a collaborator and the drug was shown to have a mean inhibitory concentration of 0.016 µg/mL. Pradofloxacin was administered at 7.5 mg/kg, PO, twice daily for the first 4 weeks of the current study and methylprednisolone acetate was administered at 5 mg/kg by intramuscular injection on week 10 (Table 1). Blood was collected for complete blood cell count, *Bartonella* spp. PCR assay, and *Bartonella* spp. IgG titer prior to administration of pradofloxacin, approximately every 2 weeks for 10 weeks, and then weekly for 4 weeks. 

## 3. Results

In the previous study, each of the cats developed detectable *Bartonella* spp. IgG titers and each had been intermittently positive for *B. henselae* DNA in blood samples after the IV inoculation. In the six weeks prior to entering this study, *Bartonella* spp. IgG was detected in every serum sample and *Bartonella* spp. DNA (4) was amplified from the blood of each cat at least once using this conventional PCR assay (Table 1). 

Vomiting, diarrhea, hyporexia, or other clinical signs were not noted by the research facility during the pradofloxacin administration period. While complete ocular examinations were not performed in this study, no clinical evidence of blindness was noted. After starting the administration of pradofloxacin, *B. henselae* DNA was not amplified in any of the blood samples, before or after administration of methylprednisolone acetate. One cat with a maximal *Bartonella* spp. IgG titer of 1:128 was intermittently seronegative after starting pradofloxacin administration (7 of 11 samples) and was still seronegative at the end of the study on Week 14. Another cat with a maximal IgG titer of 1:64 was negative the last five sample collections of the study. 

The complete blood cell data was normally distributed and so mean and standard deviation results over time are presented in Table 2. While mean neutrophil counts decreased over time during and after administration of pradofloxacin, none of the individual cats ever developed neutropenia. After administration of glucocorticoids on Week 10, 7 of 8 cats developed neutrophil counts >7 × 10^3^ on Week 12, but as noted previously in Table 1, *B. henselae* DNA was not amplified from the blood of any of the cats. None of the individual cats developed anemia or thrombocytopenia during the study. Lymphopenia was not detected in any cat prior to administration of methylprednisolone acetate. However on week 12, 6 of 8 cats had lymphopenia. 

## 4. Discussion

The pradofloxacin protocol used in this study appeared to be well-tolerated in these eight cats. While complete ocular examinations were not performed, there was no evidence of blindness during the study or by the adoptive owners of the cats. Evidence published to date suggests that the ocular safety index of pradofloxacin is high [15]. The E-test MIC of pradofloxacin for this strain of *B. henselae* suggested that the antibiotic could be effective for clearing bacteremia. 

*Bartonella henselae* DNA was not amplified from any cat after administration of pradofloxacin, even when tested for four weeks after the administration of a dose of methylprednisolone acetate. These results and the fact that two cats became seronegative as well, suggest that this pradofloxacin protocol was associated with clearance of the organism, however, these results should be interpreted carefully as results from an untreated control group are not reported as all of the cats needed to be shown to be negative prior to adoption to private homes. The neutrophilia noted after administration of the glucocorticoid was most likely related to decreased margination of neutrophils rather than activation of the infection. Our laboratory has previously had *B. henselae* infected cats that appeared to have spontaneously limited bacteremia; thus, it is possible that the cats described may have become PCR-negative without the administration of the antibiotic. In addition, in this study, the combination of culture and PCR was not used to assess for bacteremia [1,16] and this combination may be more sensitive for detection of *B. henselae* infection. 

Since cats rarely clear *Bartonella* spp. bacteremia spontaneously in the short term, the results described here are most likely from administration of pradofloxacin. Whether a treatment effect or spontaneous clearance, both are very positive results when assessed from a public health perspective. It has been proven that administration of imidacloprid topically or by collar can block the transmission of the CSU Bh1 strain [3,17]. If flea control is administered consistently to lessen reinfection, the results of this study suggest that *B. henselae* bacteremia can ultimately be limited by cats even without antibiotic therapy.

The natural route of *B. henselae* infection is via exposure to infected *C. felis*. Since there may be factors that affect *B. henselae* infection that are imparted by the fleas and since there are multiple strains of *B. henselae* and other *Bartonella* spp. that infect cats, further data with this pradofloxacin protocol should be gathered from cats infected by fleas and in cats with natural infections. 

## 5. Conclusions

Pradofloxacin at twice the label dose in the United States administered for 28 days appeared to be safe for use in this small group of cats. In addition, since the treated cats became negative for *B. henselae* DNA by PCR assay and remained negative after the administration of methylprednisolone acetate, this protocol may likely be effective for limiting bacteremia with this *Bartonella* spp. This protocol could be considered rather than relinquishment if the cat is housed in the home of a severely immune-suppressed person while additional studies are being performed in cats infected by exposure to *C. felis*. 

## Figures and Tables

**Table 1:** *Bartonella* spp. ELISA titers and PCR assay results in experimentally inoculated cats before and after the administration of pradofloxacin.

Week	−6	−3	0 ^a^	2	4	6	8	10 ^b^	11	12	13	14
Cat 1	2048	8192	2048	2048	1024	2048	2048	1024	2048	2048	1024	1024
Cat 2	256	2048	1024	1024	512	512	512	512	256	256	128	128
Cat 3	64	512	512	512	512	512	1024	512	512	128	128	256
Cat 4	256	512	128	64	0	0	64	0	64	0	0	0
Cat 5	512	512	128	128	128	256	256	256	512	1024	512	1024
Cat 6	128	128	64	0	0	0	64	64	64	0	0	0
Cat 7	16,384	2048	512	256	512	1024	1024	512	1024	256	128	512
Cat 8	4096	4096	1024	512	256	128	1024	256	256	128	128	128

The cats were inoculated with *Bartonella henselae* IV, 12 weeks prior to entering this study. Samples with the titers in bold were also positive for *B. henselae* DNA by PCR assay. ^a^ Pradofloxacin administered at 7.5 mg/kg, PO, twice daily for 28 days. ^b^ Methylprednisolone acetate administered at 5 mg/kg, IM, once.

**Table 2:** Mean and standard deviation () results of select complete blood cell count findings in 8 cats with *Bartonella henselae* infection.

Week	Hematocrit	Platelet #/µL	Neutrophil #/µL	Lymphocyte #/µL
0	38.88 (3.18)	521.1 (145.4)	7.56 (2.08)	5.8 (1.07)
2	40.5 (4.5)	538.25 (189.2)	5.3 (1.85)	5.83(1.62)
4	40.25 (3.26)	469 (198.9)	4.37 (1.4)	5.93 (2.12)
8	39.9 (3)	473.88 (110.1)	4.975 (1.5)	4.55 (1.0)
12	40.1 (3.1)	380.1 (90.1)	10.28 (1.9)	1.25 (0.2)
Normal	28 to 43	200 to 500	2 to 7	1.5 to 6

# = number × 10^3^.

## Data Availability

All data in this study are presented herein.
